# Leveraging the Electronic Health Record to Measure Resident Clinical Experiences and Identify Training Gaps: Development and Usability Study

**DOI:** 10.2196/53337

**Published:** 2024-11-06

**Authors:** Vasudha L Bhavaraju, Sarada Panchanathan, Brigham C Willis, Pamela Garcia-Filion

**Affiliations:** 1 Phoenix Children's Hospital, 1919 East Thomas Rd, Phoenix, AZ, 85016, United States, 1 6029333635, 1 6029330806; 2 Department of Biomedical Informatics, The University of Arizona College of Medicine-Phoenix, Phoenix, AZ, United States; 3 The University of Texas at Tyler, Tyler, TX, United States

**Keywords:** clinical informatics, electronic health record, pediatric resident, COVID-19, competence-based medical education, pediatric, children, SARS-CoV-2, clinic, urban, diagnosis, health informatics, EHR, individualized learning plan

## Abstract

**Background:**

Competence-based medical education requires robust data to link competence with clinical experiences. The SARS-CoV-2 (COVID-19) pandemic abruptly altered the standard trajectory of clinical exposure in medical training programs. Residency program directors were tasked with identifying and addressing the resultant gaps in each trainee’s experiences using existing tools.

**Objective:**

This study aims to demonstrate a feasible and efficient method to capture electronic health record (EHR) data that measure the volume and variety of pediatric resident clinical experiences from a continuity clinic; generate individual-, class-, and graduate-level benchmark data; and create a visualization for learners to quickly identify gaps in clinical experiences.

**Methods:**

This pilot was conducted in a large, urban pediatric residency program from 2016 to 2022. Through consensus, 5 pediatric faculty identified diagnostic groups that pediatric residents should see to be competent in outpatient pediatrics. Information technology consultants used *International Classification of Diseases, Tenth Revision* (*ICD-10*) codes corresponding with each diagnostic group to extract EHR patient encounter data as an indicator of exposure to the specific diagnosis. The frequency (volume) and diagnosis types (variety) seen by active residents (classes of 2020‐2022) were compared with class and graduated resident (classes of 2016‐2019) averages. These data were converted to percentages and translated to a radar chart visualization for residents to quickly compare their current clinical experiences with peers and graduates. Residents were surveyed on the use of these data and the visualization to identify training gaps.

**Results:**

Patient encounter data about clinical experiences for 102 residents (N=52 graduates) were extracted. Active residents (n=50) received data reports with radar graphs biannually: 3 for the classes of 2020 and 2021 and 2 for the class of 2022. Radar charts distinctly demonstrated gaps in diagnoses exposure compared with classmates and graduates. Residents found the visualization useful in setting clinical and learning goals.

**Conclusions:**

This pilot describes an innovative method of capturing and presenting data about resident clinical experiences, compared with peer and graduate benchmarks, to identify learning gaps that may result from disruptions or modifications in medical training. This methodology can be aggregated across specialties and institutions and potentially inform competence-based medical education.

## Introduction

Medical education is traditionally time-based, which presumes that learners will meet professional standards in a predetermined period of time, whereas competence-based medical education proposes an outcomes-based approach framed by competencies [1]. This latter approach requires robust data to measure outcomes and link them to competence. One such data set is the number and variety of clinical diagnoses that learners see, grounded in Kolb’s framework that emphasizes hands-on experiences and reflection as a basis for experiential learning [2].

Unlike surgical specialties requiring minimum case numbers for procedural competence, nonprocedural specialties do not endorse minimum numbers of diagnoses trainees should see to be considered competent. Literature exists on this topic across medical specialties [3-9]; however, the methods used to collect volume and variety of clinical cases are frequently incomplete, limiting their use. The variability of literature on this topic may stem from the challenge of capturing these patient experiences in a straightforward, accurate, and abstractable form.

The electronic health record (EHR) is useful for collecting patient encounters for quality improvement and business analytics. It has been incorporated in continuing professional development for practicing physicians to drive practice change [10]. Graduate medical education has also used EHR data to measure training outcomes. In 2023, Lees et al [3] performed a systematic review of the published uses of EHR data to measure competencies in medical trainees. The most common study theme identified was “trainee condition experience,” or the trainees’ involvement in patients with specific medical conditions. While study authors commonly mapped raw EHR data to diagnostic groupings and compared them with national standards or in-training examinations to identify gaps in training, there were limitations in utilization of these data. For example, studies documenting residents’ exposure to patient experiences, such as reporting the volume of diagnoses seen, often excluded important variables such as variety of diagnoses. Others examined data in aggregate rather than individualized data as is needed to link exposure and resident competence [11-16]. There are additional studies that compared individual resident clinical exposures with peer averages using the EHR and most commonly displayed these data using dashboards; however, they did not include benchmark data which provide a necessary framework to analyze the information [3,17-19].

Since 2020, the SARS-CoV-2 (COVID-19) pandemic has provided an opportunity to examine variability in diagnoses exposure for residents and to extrapolate its impact on their education. From June 2020 to February 2021, Yarahuan et al [20] noted a significant decrease in notes authored by pediatric interns on common inpatient diagnoses, on both respiratory and nonrespiratory conditions, compared with the prepandemic group. This variability resulted from the shifting prevalence of seasonal diagnoses and altered patient exposure due to practices such as “platooning” trainees for workforce preservation, shifting trainees from ambulatory to inpatient settings, and implementing telehealth [20-24]. In response, medical education leaders and learners were tasked with identifying gaps in clinical exposure compared with prepandemic standards and creating individualized learning plans; this needs assessment, however, was largely based on recall of clinical experiences in training rather than objective data [22,25,26].

In 2019, Sebok-Syer et al [27] analyzed resident and faculty feedback about the potential use of EHR data to assess gaps and inform trainees’ learning plans. The authors found that while these data may be valuable to support formative assessment practices, the data, in isolation, would portray an incomplete picture of the trainee and require context for interpretation. Meaningful analysis and presentation of EHR data are necessary in order to explore how volume and variety of clinical experiences may objectively identify gaps and inform competence.

The purpose of this pilot was to establish a process in our residency to extract meaningful EHR data for measuring clinical exposure and address associated gaps in the literature specifically to (1) develop a feasible and efficient method to capture EHR data that measure patient experiences of individual residents; (2) offer context to these data by comparing individual resident metrics to classmates and aggregated graduate residents’ data; and (3) create a visualization that provides residents and program directors with a snapshot of the volume and variety of trainees’ clinical experiences to allow quick identification of training gaps to inform focused learning plans.

## Methods

This pilot study was conducted from 2019 to 2021 in a large, urban, pediatric residency program with multiple institutional sites. To assess feasibility, we focused on ambulatory diagnoses at 1 pediatric continuity clinic site. We chose this site since it had a larger volume of general pediatric patients with fewer complex medical needs than the other continuity clinics. Subjects were limited to pediatric residents and excluded rotating residents and students as we were seeking longitudinal clinical experiences and these latter 2 groups completed only 1 block rotation in the clinic. The resident patient panels at this clinic site are a combination of patients assigned by schedulers and those recruited by residents from other settings within the health care system, such as the nursery or inpatient unit. The residents generally stay with the same faculty preceptor for 3 years and have increasing levels of autonomy during the patient visit including billing and coding; however, billing and coding are always verified by the preceptor prior to closing the encounter.

This was a retrospective analysis of EPIC (Epic Systems) EHR metadata of ambulatory clinic notes authored by pediatric residents at this clinic site from 2013 to 2020. This was true EHR metadata attached to the note, not extracted from administrative claims data. Resident data from the graduating classes of 2016, 2017, 2018, and 2019 were used as the graduation benchmarks. Data were extracted up to April 2020, representing nearly 3 years of data from the class of 2020, 2 years from the class of 2021, and 1 year from the class of 2022 (Table 1).

**Table 1.** Resident electronic health record data.

Class of	Count, n	Resident category at the time of study	Dates of data extraction	Data used for	Report distribution dates
2016	8	Graduated	7/2013-6/2016	Graduate benchmark	N/A^a^
2017	15	Graduated	7/2014-6/2017	Graduate benchmark	N/A
2018	18	Graduated	7/2015-6/2018	Graduate benchmark	N/A
2019	11	Graduated	7/2016-6/2019	Graduate benchmark for 9/2019 and 4/2020 reports	N/A
2020	18	Active	7/2017–6/2020	Individual reports and 2020 class benchmarks	4/2019, 9/2019, 4/2020
2021	19	Active	7/2018-4/2020	Individual reports and 2021 class benchmarks	4/2019, 9/2019, 4/2020
2022	13	Active	7/2019-4/2020	Individual reports and 2022 class benchmarks	9/2019, 4/2020

a N/A: not applicable.

### Ethical Considerations

The Phoenix Children’s Hospital institutional review board has determined that this project involves quality improvement and does not meet the definition of research; therefore, the approval of the institutional review board was not required and this study was deemed exempt.

### Key Stakeholders

Project stakeholders included the residency program director, residency program coordinator, and ambulatory clinic faculty preceptors. For information technology (IT) support, we engaged data analysts who recognized that graduate medical education was connected to the hospital business model and therefore supported this opportunity for improved billing and coding through EHR data analysis. Our pediatric residents were also vital participants in this pilot and were aware of its planning and rollout.

### Diagnoses Set

Five general pediatricians from 3 clinic sites determined the key diagnostic groups that pediatric residents should see to be competent for independent outpatient practice. The group created a shared mental model with inclusion and exclusion criteria. For example, high-volume diagnoses (eg, pediatric well-checks) and low-volume, yet important diagnoses (eg, gait abnormality) were included. Common, self-limited conditions (eg, upper respiratory infections) were intentionally excluded presuming that residents in our busy clinics receive adequate exposure of these during residency, and the addition of these common diagnoses in a data report may distract from the more actionable data. The final list was generated through several rounds of review and consensus. Each diagnostic group was converted to *International Classification of Diseases, Tenth Revision* (*ICD-10*) codes (Table 2).

**Table 2.** Diagnostic groups for clinical experiences of pediatric residents and associated *ICD-10^a^* codes.

Group name	*ICD-10 *codes	Notes (inclusions)
Well check	Z00.129, Z00.121	N/A^b^
Anemia	D50-D64	N/A
Constipation	K59.xx	N/A
Vomiting/diarrhea	R11.1, R11.2, R19.7, A09	N/A
Underweight/failure to thrive	R62.51	N/A
Gait problem/limp	R26.xx	In toeing, limp, genu varus, genu valgus
Genitourinary concerns	N43.xx, N47.xx, N48.xx, N90.89, K40.xx, K41.xx, Q53.xx, Q54.xx, Q55.xx	Hydroceles, phimosis, labial adhesions, hernias
Overweight/obesity, increased BMI	Z68.51-Z68.54, E66.3, E66.9, E66.09	N/A
Sexually transmitted infections	A50.0-A64, Z11.3-Z11.9	Screening and management
Asthma	J45.xx	N/A
Eczema	L30.8, L30.9, L20.9, L20.82, L20.83, L20.84, L21.1	N/A
Heart murmurs	R01.xx, I35.8, Q21.xx-Q24.xx	Functional and pathologic
Ear infections	H65.xx-H66.xx, H60.xx	Otitis media and variants, otitis externa
Urinary tract infection	N10, N30.xx, N39	N/A
Developmental delay	F80.xx, F82, F88, F89, R62.50	N/A
Behavioral/ADHD^c^	F90.0‐90.2, F90.8‐90.9, F91.0‐91.3, F91.8‐91.9, F93.0, F93.8‐94.2, F94.8‐94.9, F95, F98, F30-39.9999, F40-48.9999	Depression, anxiety, ADHD with all variants
Young women’s health	Z30.xx, N92.6, N93.9, N94.3‐97	Contraception, menstrual concerns
Headache	G43.xx-G44.xx, R51	N/A
Autism spectrum disorder	F84.xx	N/A
Genetic and chromosomal disorders	Q90-Q99.xx	N/A
Specific congenital nongenetic disorders	Q35.xx-37.xx, Q05.xx	Spina bifida and variants, cleft lip and palate
Vaccine hesitancy	Z28.xx	N/A

a *ICD-10*: *International Classification of Diseases, Tenth Revision*.

b N/A: not applicable.

c ADHD: attention-deficit/hyperactivity disorders.

### EHR Data Extraction

The project data set, including resident data, patient data, and encounter data, was abstracted from the EHR. To ensure accuracy and completeness of the data, we performed an iterative process with our IT consultants, starting with smaller subsets of data with 1 resident, to ensure that each piece of data pulled was relevant to the pilot before expanding to larger subsets and more residents. Variety was determined through *ICD-10* codes for all visit diagnoses per encounter and volume was measured as the number of unique visits. When residents authored an encounter note, they were attributed to that patient and his or her associated diagnoses.

A flat file of the EHR data was imported to Microsoft Excel and analyzed using the pivot table function. Pivot tables were created to enumerate the volume of patients for each diagnosis by individual resident. Individual resident data were aggregated to the respective class level of postgraduate year (PGY1, PGY2, or graduated) to calculate average volumes. An Excel worksheet was created for each individual resident to summarize the resident’s volume (column) and variety (rows) of clinical experiences. For comparison, the class and graduated average volumes were appended as columns. These worksheet data were used to generate the visualizations.

### Visualization and Data Report

To facilitate the assessment of training gaps, we used Microsoft Excel to translate tabular data into a radar chart (or spider graph) visualization, which is a 2D graphical method of illustrating multiple quantitative variables on axes (eg, diagnostic categories) with the same starting point. Since these data have vastly different scales on the same chart, the data were converted from raw numbers into percentages using the number of patient encounters seen for each diagnostic category (numerator) divided by the average number seen by graduated residents (denominator). The radar chart was rendered to illustrate the percentage of clinical experiences per diagnostic category for the individual resident and the class average compared with graduated residents (benchmark) as the maximal total area. Not only was this an ideal prototype for this pilot, our residents and faculty were already familiar with using radar graphs for milestone data, in which individual resident progress across a range of competencies is compared with classmates and graduates. We did trial other visualizations, including a dot plot with error bars, but found that these did not give an accurate picture because not every diagnosis was normalized at the same value.

Two comparative radar chart visualizations were created: (1) the percent volume of clinical experiences by the individual resident versus the class average and graduated residents (for individual resident review), and (2) the percent volume of clinical experiences between the aggregated classes (PGY1 and PGY2) and graduated residents (for program leadership review).

Reports with visualizations were distributed approximately every 6 months to align with semiannual reviews with clinic preceptors. Prior to distribution, residents and preceptors were educated on using the reports to stimulate discussion on learning goals.

To enable ongoing data extraction and reports, we identified 3 vital team members: the IT champion to initiate the data extraction into an Excel spreadsheet, the residency program coordinator to provide an updated list of residents at the start of each academic year and transform the raw data into individual reports with radar graph visualizations, and the clinic champion to distribute the reports and educate faculty and residents. The process was semiautomated as once the query was set up, it could be run the same way semiannually to produce a spreadsheet of every patient visit by every resident with benchmark averages calculated.

### Resident Postimplementation Surveys

Residents from the classes of 2021 and 2022 were surveyed on their individual data report readability and specific utilization for setting clinical and coding goals. The survey was homegrown by authors without validity evidence. It contained 6 multiple-choice and 2 open-ended questions and was delivered via email with instructions for completion. Results were collected anonymously. Multiple-choice questions were analyzed using frequency of responses and open-ended questions were grossly interpreted for themes and representative quotes.

## Results

We extracted information about clinical experiences for 102 residents including 52 graduated residents for the graduate benchmark and 50 active residents for individual reports and class benchmarks (Table 1). Residents from the classes of 2020 and 2021 received 3 data reports and those from the class of 2022 received 2.

**Figure 1.**
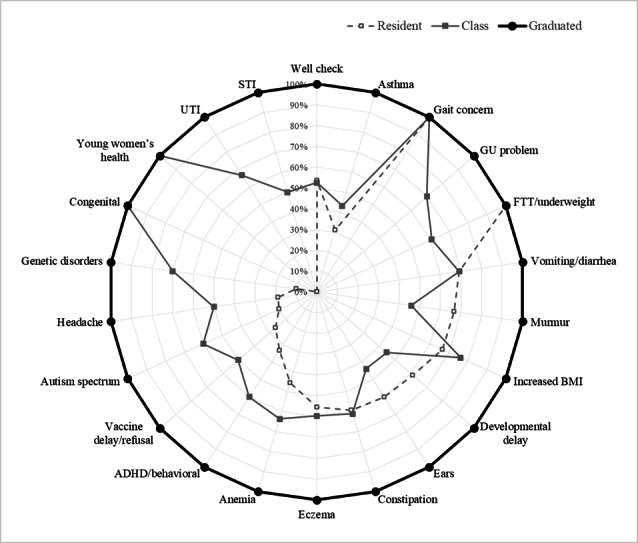
Individual resident profile with class average benchmarked against graduated residents. The diagnostic categories were deliberately placed randomly rather than ordered from high to low percentage so that residents would focus on individual categories rather than the extremes. ADHD: attention-deficit/hyperactivity disorders; FTT: failure to thrive; GU: genitourinary; UTI: urinary tract infection.

Table 3 displays data for an individual resident alongside the class and graduated residents’ averages to enable residents to follow their progress against internal benchmarks. Figure 1 uses data from Table 3 to visualize these data in a radar chart. This visualization method makes deficient areas immediately apparent to the resident and identifies which experiences must be intentionally pursued. The second semiannual report was created in Spring of the academic year when equity in clinical rotation experiences is assumed within a class and peer averages are more accurate.

**Figure 2.**
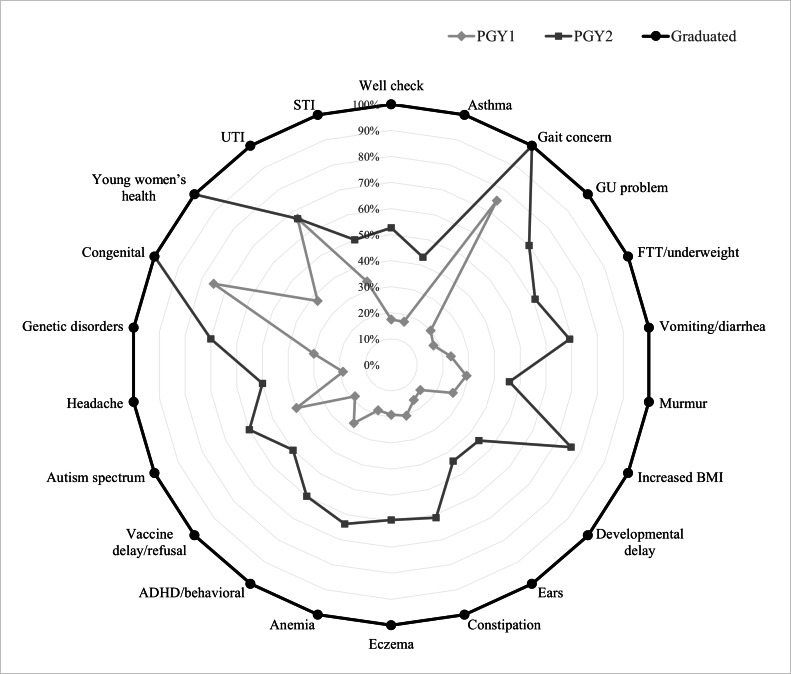
Class averages benchmarked against graduated residents. ADHD: attention-deficit/hyperactivity disorders; FTT: failure to thrive; GU: genitourinary; PGY1: postgraduate year 1; PGY2: postgraduate year 2; UTI: urinary tract infection.

The change in the class-level volumes versus graduate class volumes (Table 4) demonstrated that average volume for each diagnosis group increased progressively with each year. The visualization of this aggregated information (Figure 2) can be used by program leaders for tracking general trends in diagnoses exposure year-to-year.

**Table 3.** Sample table with average numbers of patients seen with each diagnosis in continuity clinic, by a single postgraduate year 2 (PGY2) resident, compared with the PGY2 class average and with the average numbers seen by recently graduated residents in this program.

Diagnosis description	PGY2 resident	Class average (PGY2)	Graduated residents
Well check	463	454	863
Anemia	5	7	11
Constipation	32	33	54
Vomiting/diarrhea	9	9	13
Underweight/failure to thrive	51	17	28
Gait problem/limp	7	4	4
Genitourinary concerns	23	14	20
Overweight/obesity/increased BMI	69	79	104
Sexually transmitted infections	0	3	6
Asthma	18	25	58
Eczema	26	28	47
Heart murmurs	16	11	24
Ear infections	15	11	25
Urinary tract infection	0	2	3
Developmental delay	33	24	54
Behavioral/ADHD^a^	5	9	15
Young women’s health	0	8	8
Headache	3	8	16
Autism spectrum disorder	1	3	5
Genetic and chromosomal disorders	1	7	10
Specific congenital nongenetic disorders	0	10	4
Vaccine hesitancy	10	19	38

a ADHD: attention-deficit/hyperactivity disorders.

**Table 4.** Average numbers of patients seen with each diagnosis in continuity clinic by postgraduate year class and graduated residents.

Diagnosis description	Class: PGY1^a^	Class: PGY2^b^	Graduated residents
Well check	150	454	863
Anemia	2	7	11
Constipation	11	33	54
Vomiting/diarrhea	3	9	13
Underweight/failure to thrive	5	17	28
Gait problem/limp	3	4	4
Genitourinary concerns	4	14	20
Overweight/obesity/Increased BMI	27	79	104
Sexually transmitted infections	2	3	6
Asthma	10	25	58
Eczema	9	28	47
Heart murmurs	7	11	24
Ear infections	4	11	25
Urinary tract infection	2	2	3
Developmental delay	8	24	54
Behavioral/ADHD^c^	4	9	15
Young women’s health	3	8	8
Headache	3	8	16
Autism spectrum disorder	2	3	5
Genetic and chromosomal disorders	3	7	10
Specific congenital nongenetic disorders	3	10	4
Vaccine delay/refusal	7	19	38

a PGY1: postgraduate year 1.

b PGY2: postgraduate year 2.

c ADHD: attention-deficit/hyperactivity disorders.

We surveyed graduating classes of 2021 and 2022 following implementation of this project and report distribution; 53% (17/32) of the residents responded. Of respondents, 69% (11/16) reviewed their reports with their clinic preceptors, 44% (7/16) used their reports to make clinical goals, such as “see more adolescent patients” and “increase comfort dealing with vaccine hesitancy,” and 38% (6/16) used the reports to make coding goals, such as “include Z-codes regarding counseling” and “bill more on top of well-checks.” Gross interpretation of open-ended comments showed that residents found the radar chart easy to interpret and to identify in which areas they have had less exposure than their classmates.

## Discussion

### Principal Findings

Our pilot study demonstrated an innovative method to collaborate with IT and leverage EHR data to measure and display the volume and variety of clinical experiences, relative to peers and previous program graduates, in a pediatric residency program continuity clinic. We presented the data in a functional manner to pinpoint gaps that may result from disruptive events such as the SARS-CoV-2 pandemic. For example, in Figure 1, this resident may recognize that he or she has not seen nor coded patients with young women’s health concerns, specific genetic or congenital disorders, or vaccine delay or refusal. He or she could, therefore, intentionally choose an adolescent or genetics elective as well as ensure that when seeing patients for well-checks, any additional diagnoses, such as vaccine refusal, are coded. While radar charts are not novel for representing competence in medical education, our visualization has not been previously used for this purpose [28,29]. Utilization of reports for this type of goal setting does require preceptor education and comfort with the tool, which can be achieved through faculty development and consistent use.

We believe that this methodology can be used for programs of any specialty, size, or setting. This list of diagnoses can be easily created in internal medicine or dermatology or in subspecialties such as pediatric cardiology. While the amount of resident data used in class or graduated benchmarks may be decreased in smaller-sized programs, an individual resident can still use the data to evaluate progress and set goals. Community settings may have less breadth of diagnoses than academic settings, but this substantiates the value of this innovation; if these training programs were able to review similar data from larger programs, they may be able to examine trends and program gaps that require supplementary clinical or nonclinical experiences (eg, focused didactics, external specialty rotations, or simulation). Notably, while these reports are not intended to inform summative evaluation of resident performance or any high-stakes training decisions, the data provide objectivity and specificity in resident experiences that may enrich the feedback between preceptor and resident.

The ability to access objective data on the clinical experiences of current residents compared with prior years is indispensable for program-wide or individual events that disrupt patient exposure during training, such as rotation-site closures or extended leaves of absence. Obtaining these data is feasible and can be automated with each new class. Moreover, this process may be modified to accommodate the changing landscape of medicine. New diagnoses, such as “exposure to COVID-19,” can be added to the EHR reports. Additional metadata, such as number of telehealth visits or time-to-note-completion, can also be extracted to create a comprehensive individualized “report card” of metrics, as described by Sebok-Syer et al [30], to enhance resident feedback and assessment. For these metrics, data from periodic reports (eg, semiannually), rather than from real-time dashboards, appear to be more beneficial for the recipient to set learning goals, as these data represent trends in experiences or practices over time. Furthermore, peer benchmarks are more reliable in the periodic reports, as residents in the same class will generally complete similar clinical rotations as the academic year progresses.

### Limitations

One limitation in our methodology is its dependence on accurate and complete coding of all diagnoses addressed at a patient encounter, which is often performed by residents in the clinic setting. Some diagnoses for which only discussion was required (eg, vaccine refusal) may be underrepresented and lead to gaps as noted in Figure 1. As trainees become familiar with the data, they can differentiate a lack of coding from lack of clinical exposure. In addition, a true lack of clinical exposure may be seen with important but uncommon diagnoses, and it may be harder to estimate a consistent goal number of patients to seek with these diagnoses. This may lead residents to presume that they will be less successful in managing these diagnoses should they encounter them in the future. In these cases, we rely on our faculty preceptors, when reviewing the reports with residents, to offer perspective and strategies to gain knowledge in advance or “in the moment” when encountering rare diagnoses.

Since this was a pilot study to determine feasibility, we opted to use small-group consensus to determine the diagnostic categories rather than established resources, such as certifying board examination content specifications. We also acknowledge that many diagnostic categories identified, such as urinary tract infection and asthma, are seen in other settings where the residents rotate (eg, emergency department and urgent care) thus offering an incomplete number of total exposures. In addition, there are common diagnoses, such as pain management and mental health disorders, which are not present on the list. We made these decisions as this was a pilot study limited to a single setting with a finite list of diagnoses to demonstrate proof of concept. We anticipate that expanding the list of diagnoses, designating specific categories by age groups, and implementing the process across other clinical settings would offer more representative data.

Another limitation is that the resident survey measuring acceptability and utilization of the reports was not a validated tool and was sent 1 year after the last report distribution, likely leading to recall bias and a lower rate of return. A standardized usability survey distributed in a timelier manner would have strengthened these results. The authors also recognize that while we found our business analysts, rather than clinical informaticians, to be our IT champions, this is institution specific. We encourage readers to explore all potential partnerships between IT and graduate medical education if embarking on a similar project. Finally, despite efforts to automate the process to semiannually extract data for individual resident reports, the project stalled after our 3 main team members, the residency coordinator and IT and faculty champions, left the institution within a short period of time. We were, therefore, unable to study the outcomes of learning goals set by residents and the distribution and utilization of reports for future classes. We learned that expanding teams to allow for cross-training of tasks, proper timing and transitions of responsibilities, and creating standardized operating procedures are essential for sustainability.

### Next Steps

Within our residency program, we have identified new champions to reinvigorate this process for our clinics and expand to the inpatient and emergency department settings using a set of diagnoses unique for each location. With additional data sets across varied clinical settings, we anticipate that the trends in volume and variety will be more reflective of the complete resident experience. The authors understand that comparing data internally within a program is not the ideal “gold standard” to measure competence when compared with more standardized benchmarks. Moving forward, this method can be shared across specialties and institutions to develop national benchmarks on the average volume and variety of patient encounters trainees see and provide a measure for programs to compare their experiences with others and identify gaps in training. Once these benchmarks are compared with other measures of competence, such as milestone assessment ratings, certifying examination scores, and postgraduate performance, we can better inform competence-based medical education and fill the gap in the literature on this topic.

### Conclusions

Medical education requires robust data to measure outcomes but gathering data about clinical encounters and making them meaningful can be challenging. This pilot describes a feasible method of capturing resident clinical experiences from the EHR, setting internal benchmarks using class and graduated residents’ averages, and creating a radar chart visualization that allows learners to quickly identify gaps in their training.

## Abbreviations

EHR: electronic health record
ICD-10: *International Classification of Diseases, Tenth Revision*
IT: information technology
PGY: postgraduate year
